# Nontargeted Metabolomic Profiling of a Single *Paeonia Lactiflora* Plant and its Quality Marker Identification

**DOI:** 10.1002/open.202400520

**Published:** 2025-05-24

**Authors:** Lanmeng Yan, Yanan Wu, Rui Guan, Chuanshan Jin, Rongchun Han, Jinmei Ou, Xiaohui Tong

**Affiliations:** ^1^ School of Pharmacy Anhui University of Chinese Medicine Yaohai District Hefei 230012 China; ^2^ Joint Research Center for Chinese Herbal Medicine of Anhui of IHM Anhui University of Chinese Medicine Yaohai District Hefei 230012 China; ^3^ School of Life Sciences Anhui University of Chinese Medicine Yaohai District Hefei 230012 China

**Keywords:** analytical methods, liquid chromatography, metabolomic profiling, *Paeonia lactiflora*, quality control

## Abstract

*Paeonia lactiflora* Pall., a widely used medicinal plant in traditional Chinese medicine (TCM), has diverse therapeutic properties, though its chemical composition remains inadequately explored. This study employs nontargeted metabolomics to provide a comprehensive chemical profile of a single *P. lactiflora* plant using Orbitrap high‐resolution, accurate‐mass (HRAM) mass spectrometry. A total of 214 compounds are identified, including 45 previously unreported metabolites. These compounds, spanning classes such as glycosides, flavonoids, organic acids, and triterpenes, are detected across its seven parts (leaf, petiole, stem, flower, root, xylem in root, cortex in root), with the root showing the highest chemical diversity. Six root samples collected from traditional habitats are also analyzed to propose their quality markers. From 37 compounds common to all root samples, 8 compounds are proposed as potential quality markers based on their widespread presence and abundance across traditional growing regions. This research not only enriches the chemical understanding of *P. lactiflora* but also sets the foundation for future studies on its medicinal potential and quality control in pharmaceutical applications.

## Graphical abstract

This study employs nontargeted metabolomics to provide a comprehensive chemical profile of one single *Paeonia lactiflora* plant using liquid chromatography‐mass spectrometry. A total of 214 compounds are identified, including 45 previously unreported metabolites. These compounds, spanning classes such as glycosides, flavonoids, organic acids, and triterpenes, are detected across its seven parts with the root showing the highest chemical diversity.

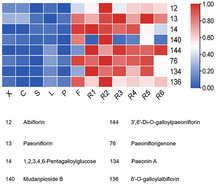

## 1 Introduction

*Paeonia lactiflora* Pall., commonly known as Chinese peony, is a perennial flowering plant widely utilized in traditional Chinese medicine (TCM). Renowned for its therapeutic properties, *P. lactiflora* has been traditionally employed to treat a variety of ailments, including inflammation, pain, and blood disorders.^[^ ^1^, ^2^, ^3^ ^]^ Despite its extensive use and established efficacy in TCM, the detailed chemical composition of *P. lactiflora* remains incompletely understood, particularly concerning its lesser‐known or unidentified compounds.^[^ ^4^ ^]^

Recent advancements in metabolomics have revolutionized the approach to study plant biochemistry.^[^ ^5^, ^6^ ^]^ Metabolomics, the comprehensive analysis of metabolites within a biological specimen offers an unparalleled opportunity to explore the chemical diversity of medicinal herbs.^[^ ^7^ ^]^ Among the various metabolomic techniques, nontargeted metabolomics stands out due to its ability to detect a wide array of metabolites without prior knowledge of their identities. This approach is particularly advantageous for identifying novel compounds that may contribute to the medicinal properties of herbs like *P. lactiflora*.

In this study, we utilized the Thermo Fisher Orbitrap Exploris 120 mass spectrometer (MS) (Thermo Fisher Scientific, USA) equipped with an electrospray ionization (ESI) source for the analysis of *P. lactiflora*. In addition to the popular analyzers such as time of flight, ion trap, and quadrupole, the Orbitrap technology represents a significant advancement in MS due to its high resolution, mass accuracy, and dynamic range. The core of the Orbitrap mass analyzer is a spindle‐shaped electrode around which ions are trapped in an electrostatic field and oscillate. These oscillations are measured to obtain mass‐to‐charge ratios with exceptional precision. The integration of ESI allows for the ionization of a wide range of metabolites, making it particularly suitable for the comprehensive analysis required in nontargeted metabolomics.

The adoption of nontargeted metabolomic approaches has been pivotal in expanding the chemical profiles of various TCM herbs. For example, Yang et al.^[^ ^8^ ^]^ employed a nontargeted metabolomic approach using ultraperformance liquid chromatography coupled with a Q Exactive MS to identify bioactive compounds in *Scutellaria baicalensis*, leading to the discovery of component differences between Kuqin and Ziqin, two commercial specifications of Scutellaria Radix. Similarly, Satria et al.^[^ ^9^ ^]^ used both gas chromatography (GC)/MS and ion‐trap time of flight (IT‐TOF)‐MS to profile the metabolites in *Ganoderma lingzhi*, uncovering the alteration of metabolites including organic acids, polyols, and fatty alcohols during eight developmental stages. Another study by Zhang et al.^[^ ^10^ ^]^ utilized nuclear magnetic resonance (NMR)‐based untargeted metabolomics to explore the chemical constituents of *Panax notoginseng*, identifying 52 components and proposing different hypoglycemic and cardiovascular protective effects due to distinct geographical indication markers.

In this study, we aim to employ a nontargeted metabolomic approach to gain a global view regarding the metabolites in *P. lactiflora*. By leveraging the capabilities of the Thermo Fisher Orbitrap Exploris 120 and advanced data analysis techniques, we seek to expand the chemical profile of *P. lactiflora* and provide chemical data for a better understanding of its medicinal potential. The high resolution and sensitivity of the Orbitrap MS, combined with the broad ionization capabilities of ESI, enable the comprehensive detection and identification of metabolites in complex biological matrices.

The findings from this research may not only contribute to the scientific knowledge of *P. lactiflora* but also pave the way for the development of novel therapeutic agents derived from this valuable medicinal herb. By uncovering the intricate metabolite composition of *P. lactiflora*, we hope to provide deeper insights into the mechanisms underlying its therapeutic effects and promote its broader application in modern medicine.

## 2 Experimental Section

### 2.1 Plant Materials and Reagents

**Figure 1:**
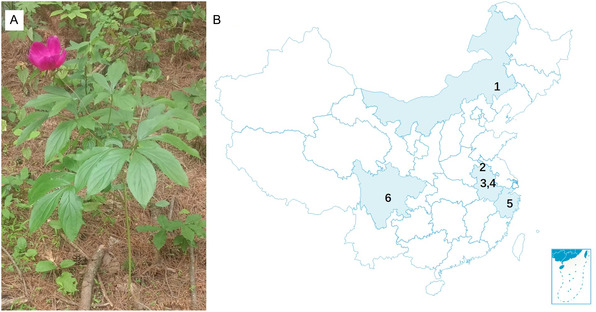
*P. lactiflora* samples for metabolomic study. A) The whole plant collected in Neimenggu, China; B) six batches of *P. lactiflora* root sampled from 1) Neimenggu, 2–4) Anhui, 5) Zhejiang, and 6) Sichuan.

One healthy *P. lactiflora* plant was sampled from Meilin Town, Chifeng City, Neimenggu, China, in 2023 and its seven parts, including leaf, petiole, stem, flower, root, xylem in root, and cortex in root, were utilized for the nontargeted metabolomic study. To assess the reliability of the chemical profile obtained, two more *P. lactiflora* plants as biological replicates were also collected from the same area in Meilin Town. Because traditional Chinese medicine uses *P. lactiflora*'s root to prepare Radix Paeoniae Alba (RPA) or Radix Paeoniae Rubra (RPR), and in order to acquire sufficient data to propose representative chemicals that reflect the quality marker of *P. lactiflora*, we sampled more roots from renowned cultivation locations and the detailed information is illustrated in **Figure** **1** and Supporting Information S1. Fresh samples were cut and freeze‐dried by a LGJ‐10 lyophilizer (Songyuan, China) for 72 h and then subjected to grinding with a mortar. The resulting fine powders which could pass through a 100‐mesh sieve were used for extraction. Taking root for example, it is easier to obtain powder from the cortex compared to a hard xylem, and therefore, all parts in the root have to be ground thoroughly and pass the sieve for subsequent analysis to avoid potentially biased results.

Acetonitrile, methanol, and formic acid of UHPLC (ultrahigh‐performance liquid chromatograph)‐MS level were provided by Merck (St. Louis, USA). Ultrapure water was produced with a PALL Cascada laboratory water system (PALL, Singapore). Twenty‐nine standard substances including paeoniflorin and albiflorin were also used for identification of interested components (Supporting Information S2).

### 2.2 Preparation of Standard and Sample Solutions

Twenty‐nine standard substances were precisely weighed and dissolved in MS grade 80% methanol, respectively, to make stock solutions with a concentration of 1.5 mg mL^−1^. Fifty milligram powder from different parts of the *P. lactiflora* plant or root batches were suspended in 1 g of 80% MS grade methanol respectively to make a mixture which was then sonicated for 1 h at 40 °C and centrifuged at 12,000 rpm for 20 min in order to extract and purify the characteristic ingredients. The supernatant was finally used for downstream analysis.

### 2.3 Chromatographic Conditions for *P. lactiflora* Samples

Samples were eluted using a Vanquish UHPLC system (Thermo Fisher, USA). The column was ALQUITY UPLC HSS T3 (2.1 mm ×100 mm, 1.8 μm) from Waters (Waters Inc., USA). The elution solutions A and B were 0.1% formic acid in water and 0.1% formic acid in acetonitrile with a flow rate of 0.3 mL min^−1^. The oven and autosampler temperatures were fixed at 30 and 10 °C, respectively. The injection volume was 3 μL. We adopted the following gradient program: 0–3 min, 8–10% B; 3–10 min, 10–32% B; 10–18 min, 32–40% B; 18–20 min, 40–90% B; 20–22 min, 90–95% B; 22–24 min, 95% B; 24–25 min, 95–8% B; 25–30 min, 8% B. Elutes were analyzed using the Orbitrap Exploris 120 system (Thermo Fisher, USA) coupled with an electrospray ionization (ESI) source which was maintained in both negative and positive ion detection mode with the parameters as follows: ion spray voltage floating at 3000 V (−) or 3000 V (+); sheath gas at 40 arb; aux gas at 7 arb; sweep gas at 1 arb; ion transfer tube temperature at 320 °C; vaporizer temperature at 325 °C; expected LC peak width set to 8 s. The scan range was set from 100 to 1500 m z^−1^.

### 2.4 Data Analysis

Structures of the identified 214 compounds were created with ChemDraw 19.0. R program and the VennDiagram package were utilized to depict identified compounds across various samples. Cluster analysis was conducted adopting Origin software (version 2021) to classify distinct *P. lactiflora* samples based on all identified chemicals. For quality marker identification, with the help of SIMCA (version 14.1), partial least squares discriminant analysis (PLS‐DA) models were established in both negative and positive modes with VIP values (>1) as well as *p* values (<0.05) monitored for robust biomarkers. Concerning nontargeted metabolomic interpretation, Compound Discoverer (version 3.3) and Xcalibur software (version 4.1.31) as well were adopted by following the manufacturer's instructions (Thermo Fisher, USA). To interrogate the obtained data and find reliable compounds, we took advantage of both online and local databases which included mzVault (Custom mzVault Library, Bamba lab 598 polar metabolites stepped NCE 10 30 45, LipidBlast‐VS68‐Neg, LipidBlast‐VS68‐Pos, PFAS_CFM_specLibrary_Duke), ChemSpider (BOC Sciences, Nature Chemical Biology, Phenol – Explorer, PlantCyc, NPAtlas), mzCloud, Arita Lab 6549 Flavonoid Structure Database, Natural Products Atlas 2023‐06, NIST‐sdf, and Chemical List PFASSTRUCT −2022‐04‐20.

## 3 Results

### 3.1 Identification of Chemicals from Different Organs of *P. lactiflora*

As a result, we identified or tentatively characterized a total of 214 compounds from both negative and positive modes in a single *P. lactiflora* plant. The total ion chromatogram (TIC) chromatograms for the single *P. lactiflora* plant are demonstrated in Supporting Information S3 which comprised negative and positive modes of leaf, petiole, stem, flower, root, xylem in root, and cortex in root. For a better illustration, we also labeled all detected compounds on the TIC chromatograms. The corresponding chemical structures of the 214 compounds are illustrated in **Figure** **2**. The compounds could be categorized into several groups including glycosides, flavonoid compounds and their derivatives, organic acids, ketones and esters, alcohols, phenols, tannins, triterpenes, iridoids and so forth. In Supporting Information S4, detailed information regarding the formula, calculated molecular weight, MS fragmentation, retention time, and target parts where corresponding compounds are detected is presented. Compound Discoverer automatically annotates compounds when the patterns of the respective MS fragments match its built‐in database. In addition, standard procedure for identification of interested compounds, using paeoniflorin as an example (No 13 in the compound list presented in Supporting Information S4), was also concluded in Supporting Information S5.

Figure 2Chemical structures of the 214 identified compounds in *P. lactiflora*.

**Figure:**
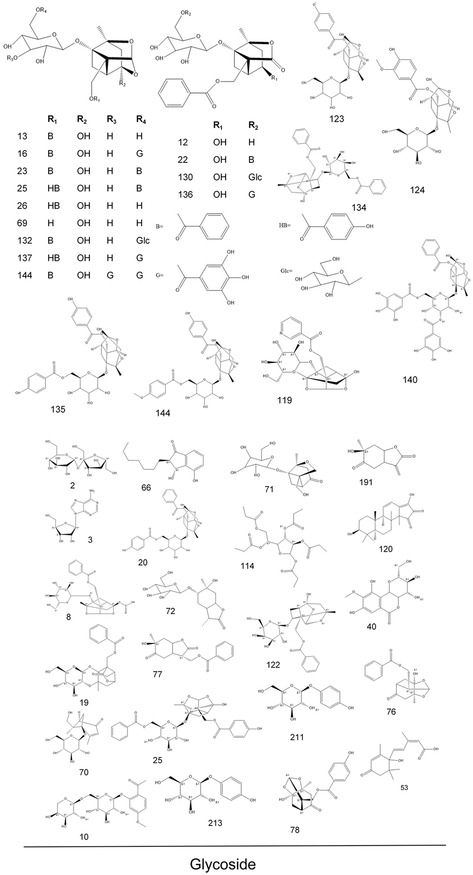

**Figure:**
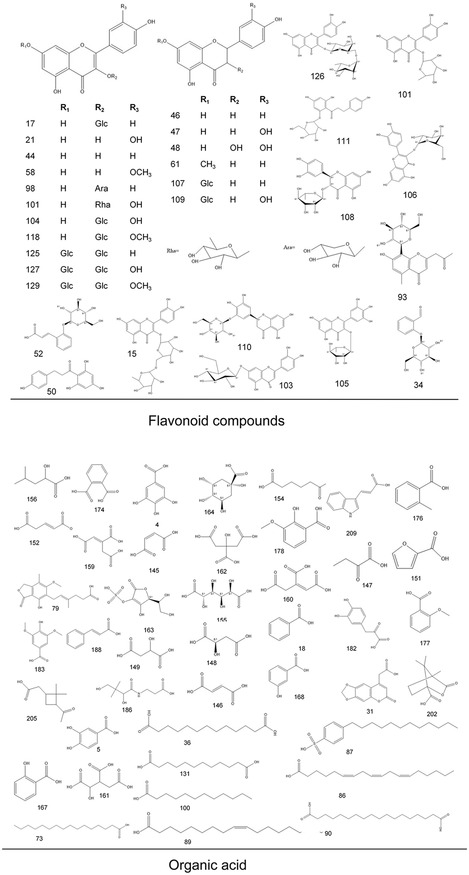

**Figure:**
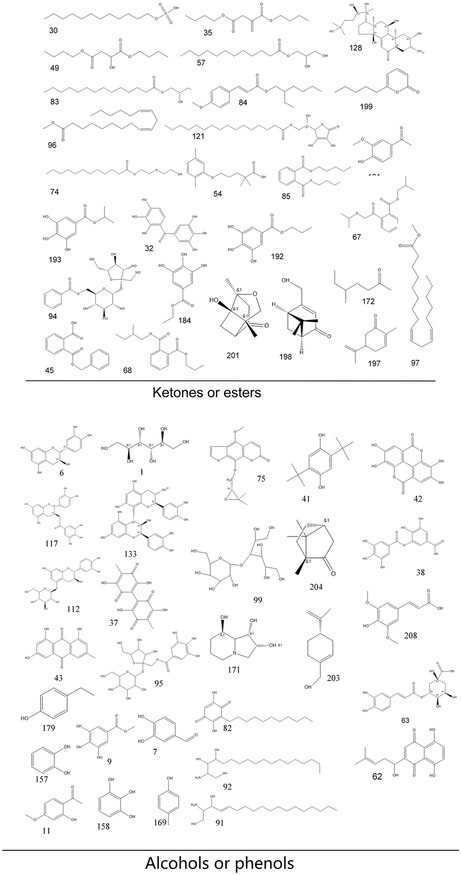

**Figure:**
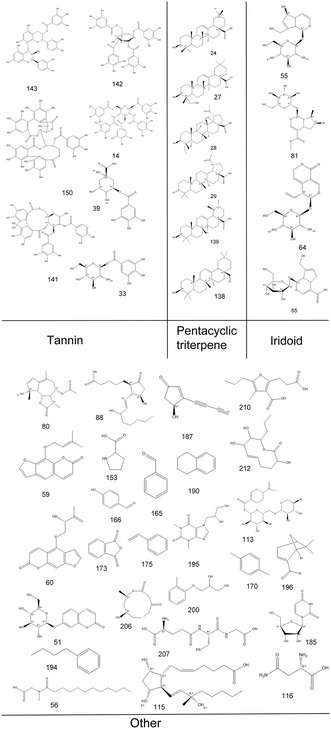

For compounds that have isomers, they may be distinguished by the characteristic MS fragmentation patterns reported in literature, or may be compared with the retention times of reference standards. Taking paeoniflorin and albiflorin for example, the monoisotopic mass for both compounds is 480.16316. By using standard compounds, distinct retention times are confirmed. Additionally, MS fragmentation also reveals different fragment ions which help us to pinpoint the two isomers (Supporting Information S4).

### 3.2 Discovering Quality Marker for the Root of *P. lactiflora*

In China, due to the long history of *P. lactiflora*, the application of its root is sophisticated. Root of *P. lactiflora* can be regarded as RPA or RPR depending on different processing methods applied. On the other hand, paeoniflorin is currently the only marker component recognized by several official pharmacopeias to assess the quality of RPA or RPR.^[^ ^11^, ^12^, ^13^ ^]^ With more than 200 chemicals identified from *P. lactiflora* and versatile derivatives closely related to paeoniflorin confirmed, we set out to propose quality markers by combing the data on hand to reflect genuine situation that the markers are present in all major habitats of *P. lactiflora* with considerable concentrations.

**Figure 3:**
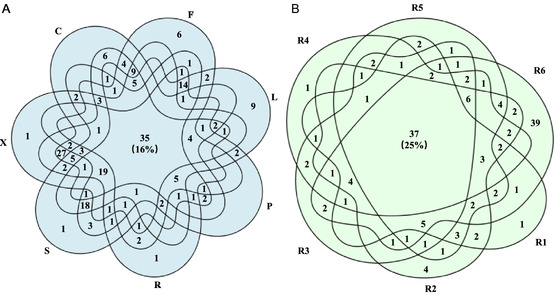
Intersections for the chemicals detected in distinct samples. A) Number of shared compounds among the 7 tested samples of a single *P. lactiflora* plant. B) Number of shared compounds among the 6 root samples collected from traditional habitats. For a concise presentation, numbers equal 0 in the intersections are intentionally left blank.

**Figure 4:**
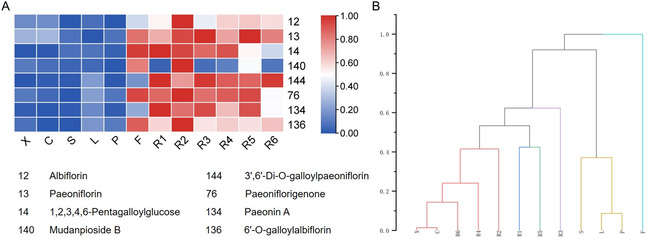
A) Heatmap illustrating the peak areas for the designated 8 quality markers for *P. lactiflora*. B) Cluster analysis to classify *P. lactiflora* samples based on identified compounds.

Based on the data originated from the seven parts of the single *P. lactiflora* plant, it was found that 35 compounds were detectable from all tested samples (**Figure** **3**A). The second largest intersection contains 27 chemicals (13%) belonging to X∩R∩C and this makes sense because from the perspective of plant anatomy, xylem and cortex are the two connected parts in the root. In order to obtain reliable quality markers for *P. lactiflora*, 146 compounds that were detectable from the root (Supporting Information S4) were selected and at the same time, information concerning the presence of these chemicals in individual root samples (R1–R6) was also integrated to create a Venn diagram. As a result, 37 known compounds (25%) that were shared by all 6 root samples were retrieved (Figure 3B). A representative list of compounds that are shared by the traditional habitats of *P. lactiflora* is vital to propose its quality marker. At the same time, abundance, in the form of peak areas, is also indispensable. We then extracted the top 75 compounds from both negative and positive modes, respectively, using the built‐in application PLS‐DA in Compound Discoverer. Since ideal quality marker should be present in all root samples collected from the aforementioned renowned habitats, we reduced the number from 150 to 14 by creating an intersection. The software SIMCA was subsequently used to test the validity of these candidates and resulted in 8 compounds with their VIP values >1 and *p* values <0.05. Finally, by taking into consideration the previous reports,^[^ ^14^, ^15^, ^16^, ^17^ ^]^ we proposed 8 compounds as the quality marker of *P. lactiflora*: paeoniflorin, albiflorin, mudanpioside B, paeonin A, 1,2,3,4,6‐pentagalloyl glucose, paeoniflorigenone, 3′,6′‐di‐O‐galloylpaeoniflorin, and 6′‐O‐galloylalbiflorin. The abundance of the 8 chemicals is presented in **Figure** **4**A, and judged by the peak areas, the abundance of the compounds in root samples was usually higher than that of the aerial parts. Furthermore, we started to sort respective *P. lactiflora* samples from the perspective of all 214 identified compounds using the software Origin. It turned out that all the root samples were grouped together, while aerial parts (excluding flower) and flower comprised the other two clusters (Figure 4B). The three clusters are meaningful in terms of representing the natural relationship among plant organs from the perspective of accumulated metabolites, suggesting the robustness of our findings in this study.

## 4 Discussion

This study represents a pioneering effort in the nontargeted metabolomic profiling of *P. lactiflora* using advanced Orbitrap high‐resolution, accurate‐mass (HRAM) MS technology. To our knowledge, this is the first attempt to systematically identify compounds from a single plant of *P. lactiflora* utilizing such a comprehensive approach. Previous research has largely focused on targeted analysis of specific compounds or parts of the plant,^[^ ^18^, ^19^, ^20^ ^]^ but our nontargeted strategy allowed for the detection and identification of a much broader spectrum of metabolites across various plant parts.

Our findings significantly expand the existing chemical profile of *P. lactiflora*. We identified a total of 214 compounds, 45 of which were first reported in *P. lactiflora*, enriching the database of known metabolites within this species. This includes a diverse range of chemical classes such as glycosides, flavonoids, organic acids, ketones, esters, alcohols, phenols, tannins, triterpenes, and iridoids. Notably, this study uncovered 37 compounds present in all root samples from traditional cultivation locations. With additional information including abundance and previous reports, we proposed 8 compounds as the quality markers for *P. lactiflora*. Exploring additional compounds from *P. lactiflora* could unlock novel therapeutic potentials, as this plant is already known in traditional Chinese medicine for its diverse pharmacological activities. Given that many medicinal plants exert their effects through multiple signaling pathways, identifying more bioactive compounds from *P. lactiflora* may reveal synergistic mechanisms, leading to enhanced efficacy in treating complex diseases. By unraveling these compounds, we can better understand how they interact at the molecular level, ultimately contributing to more holistic and harmonious treatments in clinical practice.

The discovery of the 45 newly identified compounds in *P. lactiflora* not only enriches its chemical understanding but also highlights potential new bioactive substances that may contribute to its medicinal properties. These findings pave the way for further pharmaceutical studies to explore their therapeutic potentials. Future research could focus on the bioactivity assays of these compounds to determine their efficacy and mechanisms of action. Additionally, expanding this metabolomic approach to other species within the genus *Paeonia* could provide a broader view of the metabolic diversity and its implications for traditional medicine.

While our report offers a comprehensive chemical profile of *P. lactiflora*, it is not without limitations. The reliance on a single plant sample may not fully represent the chemical variability present across different growing conditions and geographical locations. Furthermore, the identification of compounds based on MS alone may result in ambiguities that require confirmation through additional techniques such as NMR spectroscopy. This study serves as a stepping‐stone for the following quality evaluation of the crude drug, compound extraction, exhaustive chemical characterization, and pharmacological applications.

## 5 Summary

This study employs nontargeted metabolomics using Orbitrap HRAM MS technology to comprehensively analyze the chemical profile of *P. lactiflora*, a plant traditionally used in Chinese medicine. The analysis identified 214 compounds, including 45 novel ones, across various plant parts, such as glycosides, flavonoids, organic acids, and triterpenes. The root, commonly used in medicine, was found to contain 37 compounds common to all samples. Based on their abundance and presence across regions, 8 compounds were proposed as quality markers. These findings enhance the understanding of *P. lactiflora*'s medicinal potential and lay the groundwork for future pharmacological studies.

## Conflict of Interest

The authors declare no conflict of interest.

## Supporting information

Supplementary Material

## Data Availability

The data that support the findings of this study are available from the corresponding author upon reasonable request.
