# Supplementary material for: Nontargeted Metabolomic Profiling of a Single Paeonia Lactiflora Plant and its Quality Marker Identification
Source: ChemistryOpen. 2025 May 24;14(8):e202400520. doi: 10.1002/open.202400520 (PMC12368873; doi:10.1002/open.202400520)
Supplement: Supplementary file 1 — Supplementary Material [file OPEN-14-e202400520-s001.pdf]

Supplementary material 1 Sample information from traditional cultivation locations

Sample location	Sample tags	Date of collection	Coordinates
Kalaqin district, Chifeng	L1 (leaf)<br>P1 (petiole)<br>S1 (stem)<br>F1 (flower)<br>R1 (root)<br>X1 (xylem in root)<br>C1 (cortex in root)	28 Sep, 2023	LO 118.2826;<br>LA 41.6225
Qiaocheng district, Bozhou	R2 (root)	3 Oct, 2023	LO 115.7091;<br>LA 33.8702
Yaohai district, Hefei	R3 (root)	10 Oct, 2023	LO 117.3976;<br>LA 31.9441
Yaohai district, Hefei	R4 (root)	10 Oct, 2023	LO 117.3976;<br>LA 31.9441
Pan’an county, Jinhua	R5 (root)	1 Oct, 2023	LO 120.2146;<br>LA 28.5637
Zhongjiang county, Deyang	R6 (root)	5 Oct, 2023	LO 104.6035;<br>LA 31.0151

Supplementary material 2 Standard substances applied and their specifications

No	Name of Chemical	Specification	Manufacturer
1	Paeoniflorin	98% purity	Merck (St. Louis, USA)
2	Albiflorin	99% purity	Macklin (Shanghai, China)
3	Benzoylpaeoniflorin	99% purity	Desite (Chengdu, China)
4	Benzoylalbiflorin	97% purity	Macklin (Shanghai, China)
5	Benzoyloxypaeoniflorin	98% purity	Desite (Chengdu, China)
6	Mudanpioside C	98% purity	Yuanye (Shanghai, China)
7	Betulinic acid	98% purity	Yuanye (Shanghai, China)
8	Oleanolic acid	97% purity	Macklin (Shanghai, China)
9	23-Hydroxybetulinic acid	98% purity	Merck (St. Louis, USA)
10	Hederagenin	94% purity	Yuanye (Shanghai, China)
11	Galloylpaeoniflorin	95% purity	Macklin (Shanghai, China)
12	1,2,3,4,6-Pentagalloylglucose	96% purity	Macklin (Shanghai, China)
13	Oxypaeoniflorin	98% purity	Macklin (Shanghai, China)
14	Paeonol	99% purity	Desite (Chengdu, China)
15	Astragalin	98% purity	Macklin (Shanghai, China)
16	Lactiflorin	98% purity	Macklin (Shanghai, China)
17	3,4-Dihydroxybenzaldehyde	99% purity	Yuanye (Shanghai, China)
18	Protocatechuic acid	98% purity	Merck (St. Louis, USA)
19	Gallic acid	99% purity	Macklin (Shanghai, China)
20	Methyl gallate	98% purity	Macklin (Shanghai, China)
21	Paeoniflorin sulfite	98% purity	Macklin (Shanghai, China)
22	Catechin	95% purity	Macklin (Shanghai, China)
23	Paeonolide	95% purity	Merck (St. Louis, USA)
24	Rutin	99% purity	Desite (Chengdu, China)
25	Sucrose	97% purity	Merck (St. Louis, USA)
26	D-Mannitol	99% purity	Merck (St. Louis, USA)
27	Adenosine	99% purity	Merck (St. Louis, USA)
28	Benzoic acid	99% purity	Merck (St. Louis, USA)
29	Quercetin	95% purity	Merck (St. Louis, USA)

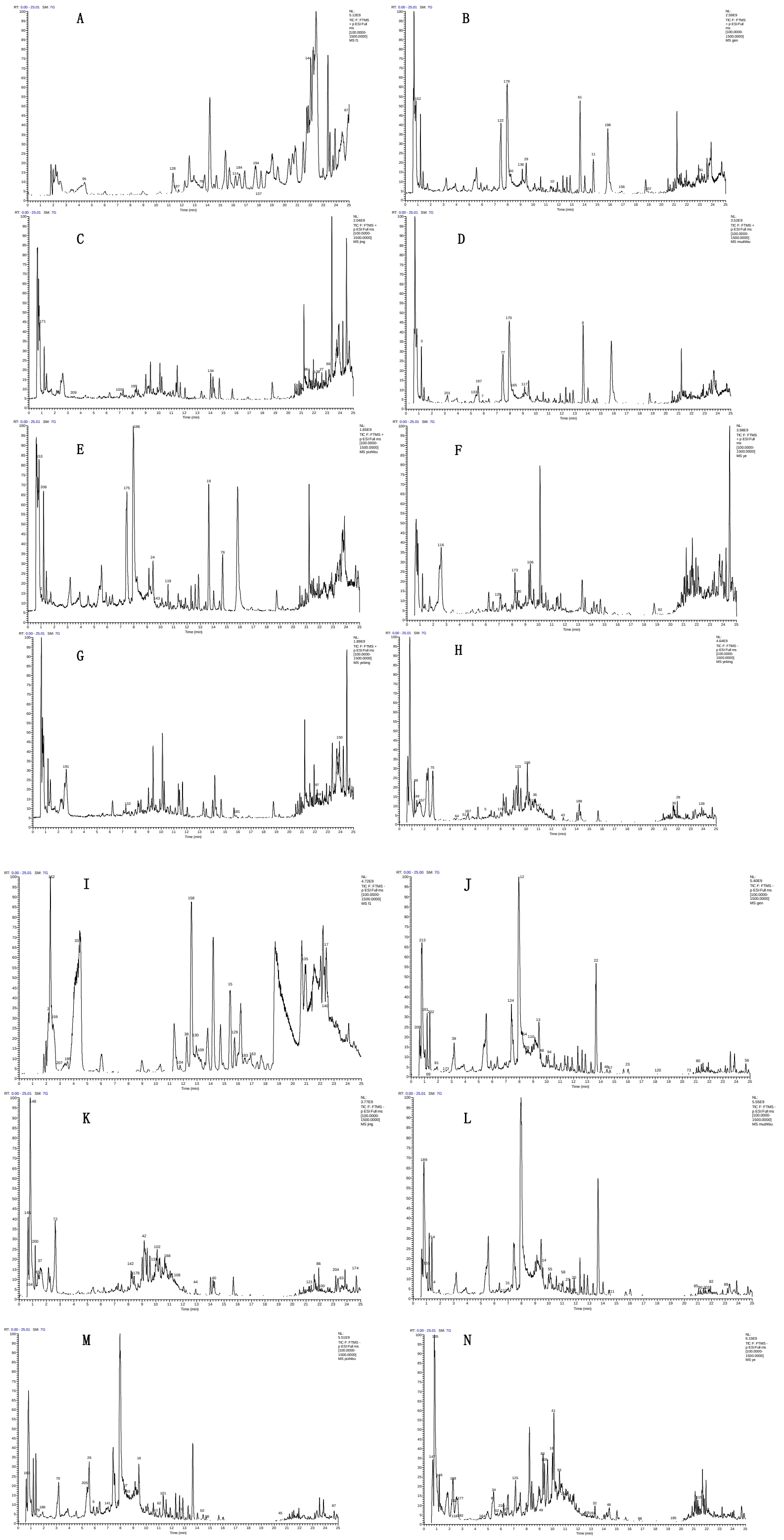

Supplementary material 3 Total ion chromatogram chromatograms for a single *P. lactiflora* plant. Positive mode of flower (A), root (B), stem (C), xylem in root (D), cortex in root (E), leaf (F), petiole (G) and negative mode of petiole (H). Negative mode of flower (I), root (J), stem (K), xylem in root (L), cortex in root (M), and leaf (N). Numbers in the diagram represent respective compounds identified in *P. lactiflora*.

Supplementary material 4 Compounds identified from different organs of *P. lactiflora* by orbitrap mass spectrometry

No.	Name	Formula	Mass Error [ppm]	Calc. MW	m/z	Adducts	MS^E Fragmentation	RT [min]	Source
1*	D-Mannitol	$C_6H_{14}O_6$	0.56	182.07914	183.0861	+H	183.1017,165.0912,147.0805,117.0698,83.0493,69.0335,55.0178,43.0179	0.98	C,R,X
2*	Sucrose	$C_{12}H_{22}O_{11}$	0.01	342.11622	341.1089	-H	341.1091,179.0562,119.0351,89.0245,71.0139,59.0139	2.24	C,F,L,P,R,S,X
3*	Adenosine	$C_{10}H_{13}N_5O_4$	0.49	267.09688	268.1041	+H	268.1041,136.0617	1.17	C,F,P,R,S,X
4*	Gallic acid	$C_7H_6O_5$	0.4	170.02159	169.01431	-H	169.0144,125.0246,107.0143,97.0297,81.0347,69.0347,51.0242	1.61	R,S,X
5*	Protocatechuic acid	$C_7H_6O_4$	1.34	154.02682	153.01954	-H	153.0196,109.0297,91.0193,81.0348,65.0396	6.83	P,S
6*	Catechin	$C_{15}H_{14}O_6$	0.73	290.07925	289.07195	-H	289.0723,245.0822,203.0717,179.0353,151.0404,123.0454,109.0297,97.0296	5.9	C,F,R,X
7*	3,4-Dihydroxybenzaldehyde	$C_7H_6O_3$	1.69	138.03193	139.0392	+H	139.0391,111.0441,93.0335,65.0386	5.87	C,F,R,X
8*	Paeoniflorin sulfite	$C_{23}H_{28}O_{13}S$	0.79	544.12549	567.1861	+Na	249.0761,179.0712,151.0759,105.0336,79.0541	13.64	C,F,R,X
9*	Methyl gallate	$C_8H_8O_5$	0.26	184.03722	183.02994	-H	183.0302,168.0067,124.0168	5.4	C,F,L,P,R,S,X
10*	Paeonolide	$C_{20}H_{28}O_{12}$	0.23	460.15818	461.16543	+H	461.1436,443.1332,179.0703,151.0754,121.0285,81.0335,69.0335	11.43	C,R,X
11*	Paeonol	$C_9H_{10}O_3$	4.55	166.06375	167.0704	+H	167.0704,149.0598,121.0647,43.0179	15.87	C,P,R,S,X
12*	Albiflorin	$C_{23}H_{28}O_{11}$	0.78	480.16316	479.15646	-H	479.15701, 449.14130,327.11002,283.08313	7.52	C,F,L,P,R,X
13*	Paeoniflorin	$C_{23}H_{28}O_{11}$	0.98	480.16316	479.15625	-H	449.14734,327.10886,165.05589,121.02968	10.14	C,F,L,P,R,X
14*	1,2,3,4,6-Pentagalloylglucose	$C_{41}H_{32}O_{26}$	1.2	940.11931	939.11208	-H	939.1127,769.0909,617.0790,465.0688,169.0146,125.0247	9.56	C,F,L,P,R,S,X
15*	Rutin	$C_{27}H_{30}O_{16}$	2.26	610.15477	609.14749	-H	609.1474,447.0936,283.0251,255.0303,227.0341,151.0034	8.079	F,L
16*	Galloylpaeoniflorin	$C_{30}H_{32}O_{15}$	2.24	632.17554	631.16826	-H	631.1680,613.1572,491.1200,399.0934,313.0573,169.0146,125.0247	9.5	C,F,L,R,X
17*	Astragalin	$C_{21}H_{20}O_{11}$	1.27	448.10169	447.09385	-H	447.0941, 284.0333, 255.0305, 227.0355	22.45	F,L,P,S
18*	Benzoic acid	$C_7H_6O_2$	1.37	122.03695	121.02967	-H	121.0297,94.0300,77.0398,57.0347	10.07	C,L,P,R,S,X
19*	Lactiflorin	$C_{23}H_{26}O_{10}$	0.3	462.15274	463.15997	+H	463.1608,445.1498,323.1131,179.0704,161.0599,151.0755,105.0335,69.0336	13.68	C,R,X
20*	Mudanpioside C	$C_{30}H_{32}O_{13}$	-0.5	600.18399	599.1776	-H	599.1776,569.1678,477.1423,165.0556,137.0246,121.0298,93.0348	11.83	C,R,X
21*	Quercetin	$C_{15}H_{10}O_7$	1.26	302.04303	301.03574	-H	301.0358,178.9988,151.0039,121.0297,107.0141	12.81	C,L,R,X
22*	Benzoylalbiflorin	$C_{30}H_{32}O_{12}$	2.34	584.19074	583.18347	-H	553.1721,431.1349,165.0560,121.0297,77.0398	13.96	C,L,P,R,S,X
23*	Benzoylpaeoniflorin	$C_{30}H_{32}O_{12}$	1.65	584.19074	629.18859	+HCOO	583.1830,461.1477,387.1080,310.2933,121.0297	16.97	C,L,P,R,S,X
24*	Oleanolic acid	$C_{30}H_{48}O_3$	0.72	456.36067	457.36807	+H	457.1141,439.1036,313.0702,183.0293,153.0180,123.0441,109.0285,81.0335	9.42	C,R,X
25*	Benzoyloxypaeoniflorin	$C_{30}H_{32}O_{13}$	0.47	600.18457	599.1775	+H	599.1775,581.1666,447.1300,431.1356,281.0670,239.0560,179.0351,137.0246,121.0297	11.41	C,R,X
26*	Oxypaeoniflorin	$C_{23}H_{28}O_{12}$	1.4	496.15877	495.15151	-H	495.1512,465.1393,281.0656,177.0556,165.0560,137.0246,93.0348,59.0139	5.52	C,F,L,R,X
27*	Hederagenin	$C_{30}H_{48}O_4$	0.72	472.3556	473.36288	+H	473.3625,427.3573,409.3457,205.1589,189.1640,95.0855	22.95	L,P,S
28*	23-Hydroxybetulinic acid	$C_{30}H_{48}O_4$	1.69	472.35606	471.34876	-H	471.3487,432.3911,329.0593,256.2563,124.0961,86.2490	21.93	C,L,P,R,S,X
29*	Betulinic acid	$C_{30}H_{48}O_3$	2.62	456.3598	457.1166	+H	457.1125,439.1039,153.0185,123.0442,109.0283,81.0334	9.44	C,R,X
30	Dodecyl sulfate	$C_{12}H_{26}O_4S$	1.21	266.1555	265.14822	-H	269.1482,211.5244,173.2067,96.9603,79.9576	21.74	C,L,P,R,S,X
31	(6-Oxo-6H-[1,3] dioxolo[4,5-g]chromen-8-yl)acetic acid	$C_{12}H_8O_6$	1.47	248.03245	247.02517	-H	247.0233,221.0467,203.0352,175.0402,147.0453,119.0504,71.7099	7.07	C,R,X
32	Exifone	$C_{13}H_{10}O_7$	1.76	278.04314	277.03586	-H	277.0358,249.0409,190.0272,129.0196,85.0296	13.38	L,P,S
33	Glucogallin	$C_{13}H_{16}O_{10}$	-0.07	332.07432	331.06704	-H	331.0674,271.0466,211.0250,169.0143,151.0038,125.0246,71.0138,59.0137	4.38	C,F,L,P,R,S,X
34	Helicin	$C_{13}H_{16}O_7$	1.04	284.0899	283.08262	-H	283.0828,264.9449,239.0930,195.1029,177.0924,149.0974,97.0297,69.0348	5.56	L,P,S
35	Dibutyl itaconate	$C_{13}H_{22}O_4$	1.52	242.15218	241.1449	-H	187.1340,167.1087,149.0982,137.0972,63.9856	8.4	C,R,X

36	Brassylic acid	$C_{13}H_{24}O_4$	0.99	244.1677	243.16042	-H	243.1242,225.1139,183.1028,139.1131,59.0140	10.84	L,P,S
37	Osporein	$C_{14}H_{10}O_8$	1.8	306.03812	305.03086	-H	305.1396,169.0144,124.0168,78.0115,60.8550	21.13	C,L,P,R,S,X
38	Digallic acid	$C_{14}H_{10}O_9$	0.95	322.03279	321.02551	-H	321.1348,277.1448,168.0066,124.0168,78.0115	12.7	C,F,L,P,R,S
39	Theogallin	$C_{14}H_{16}O_{10}$	-0.29	344.07425	343.06696	-H	343.1404,227.8687,181.0870,151.0767,136.0530,109.0660,71.0139	3.17	C,R,X
40	Bergenin	$C_{14}H_{16}O_9$	0.64	328.07964	327.07236	-H	327.2183,291.1973,229.1449,211.1344,171.1031,85.0298	14.27	C,F,L,P,R,S,X
41	Beta-D-Ethyl glucuronide	$C_8H_{14}O_7$	1.45	222.07428	221.06701	-H	221.1545,176.8604,121.0298,99.9259,83.9310	10.1	L,P
42	Ellagic acid	$C_{14}H_6O_8$	1.9	302.00684	300.99956	-H	300.9994,283.9969,229.0142,201.0194,173.0246,145.0296,65.4725	9.2	C,F,L,P,R,S,X
43	Archin	$C_{15}H_{10}O_5$	2.09	270.05339	269.04611	-H	269.1398,251.1295,225.1499,209.1186,165.1288,99.0454,59.0139	12.77	L,P,S
44#	Kaempferol	$C_{15}H_{10}O_6$	1.23	286.04809	285.04081	-H	285.0044,213.0191,185.0246,151.0038,133.0297,107.0141	12.96	C,F,L,P,R,S,X
45	Monobenzyl phthalate	$C_{15}H_{12}O_4$	1.64	256.07398	255.0667	-H	255.0665,213.0559,171.0456,151.0039,107.0140,83.0139	20.53	C,L,P,R,S,X
46	Naringenin	$C_{15}H_{12}O_5$	1.59	272.06891	271.06163	-H	271.0617,151.0038,119.0503,107.0141,93.0349	14.46	C,F,L,P,R,S,X
47	Eriodictyol	$C_{15}H_{12}O_6$	1.83	288.06392	287.05664	-H	287.0566,259.0616,243.0666,125.0246,83.0140	11.03	C,F,L,P,R,S,X
48#	Taxifolin	$C_{15}H_{12}O_7$	1.96	304.0589	303.05163	-H	303.0517,285.0409,177.0195,125.0246,83.0141	9.58	C,F,R,X
49	Dibutyl malate	$C_{12}H_{22}O_5$	1.32	246.14705	245.13977	-H	245.1402,227.1293,209.1187,201.1136,127.1131,58.0061	9.14	L,P
50#	Phloretin	$C_{15}H_{14}O_5$	1.62	274.08457	273.07729	-H	273.0774,167.0353,123.0454,81.0348	1455	C,R,X
51	Skimmin	$C_{15}H_{16}O_8$	1.28	324.08493	323.07768	-H	323.1352,119.0351,101.0246,89.0246,71.0140,59.0139	5.04	C,F,L,P,R,S,X
52	Melilotoside	$C_{15}H_{18}O_8$	0.49	326.10033	325.09303	-H	325.0942,163.0403,119.0504	5.69	F,L,P,S
53	(±)-Abscisic acid	$C_{15}H_{20}O_4$	2.19	264.13674	263.12945	-H	219.1394,204.1158,201.1287,153.0924	8.56	C,R,X
54	Gemfibrozil	$C_{15}H_{22}O_3$	1.22	250.1572	249.14992	-H	249.0807,96.9604,79.9577	8.41	C,R
55	Aucubin	$C_{15}H_{22}O_9$	0.07	346.12641	345.11898	-H	197.0825,164.04834,139.0399	10.09	C,F,L,P,R,S,X
56	Lauroylsar cosine	$C_{15}H_{29}NO_3$	0.8	271.21496	270.2077	-H	95.5378,90,3258,88.4445	24.99	R
57	DL-α-Laurin	$C_{15}H_{30}O_4$	1.55	274.21483	273.20756	-H	273.0772,167.0352,123.0454,81.0247	14.46	C,R,X
58#	Isorhamnetin	$C_{16}H_{12}O_7$	2.42	316.05907	315.05178	-H	315.0151,299.9916,216.0074	11.08	C,R,X
59	Imperatorin	$C_{16}H_{14}O_4$	2	270.08975	269.08247	-H	287.0562,259.0620,177.0558,125.0246	12.77	L,P,S
60	Pangelin	$C_{16}H_{14}O_5$	0.21	286.08418	287.09146	+H	119.0858,137.0963,105.0337	8.39	C,R,X
61	Sakuranetin	$C_{16}H_{14}O_5$	0.21	286.08418	287.09146	+H	287.0930,67.9076	13.62	C,R,X
62	Shikalkin	$C_{16}H_{16}O_5$	1.87	288.10031	287.09304	-H	288.9352,217.0884,190.8624	10.99	C,F,L,P,R,S,X
63	Chlorogenic acid	$C_{16}H_{18}O_9$	0.09	354.09511	353.08772	-H	353.1432,116.9288,100.9338,84.9389	23.71	C,L,P,S
64	Gentiopicrin	$C_{16}H_{20}O_9$	-0.3	356.11062	355.10335	-H	194.0548,148.0533,176.0435	4.55	L,P,S
65	Geniposidic acid	$C_{16}H_{22}O_{10}$	0.28	374.1214	373.11412	-H	211.0614,193.0506,149.0609,131.0502	1.53	L,P,S
66	Paecilocin D	$C_{16}H_{22}O_3$	2.83	262.15764	263.16491	+H	227.8174,89.0600,69.0337	23.12	C,L,P,R,S,X
67	Dibutyl phthalate	$C_{16}H_{22}O_4$	2.42	278.15248	301.14171	+Na	147.9484,121.9626,108.8647,103.4083,57.0700	24.89	F
68	2-methyl butyl propyl phthalate	$C_{16}H_{22}O_4$	2.42	278.15248	301.14171	+Na	149.0235,57.0699	22.45	C,F,L,P,R,S,X
69	8-Debenzoylpaeoniflorin	$C_{16}H_{24}O_{10}$	0.33	376.13707	421.13528	+HCOO	375.1277,345.1197,195.0662,139.0401	1.17	C,F,L,P,R,X
70#	Mudanpioside F	$C_{16}H_{24}O_8$	0.39	344.14725	389.14547	+HCOO	343.1399,181.0872,151.0766	3.17	C,R
71#	1-O-β-D-glucopyranosylpaeonisuffron	$C_{16}H_{26}O_9$	0.17	362.15800	361.1510	-H	361.1510,59.0140	1.79	C,R
72#	6-O-β-D-glucopyranosyl lactinolide	$C_{16}H_{26}O_9$	-0.03	362.15767	407.15589	+HCOO	351.1505,71.0139,59.0139	2.66	L,P,S
73	Palmitic acid	$C_{16}H_{32}O_2$	1.45	256.2406	255.23333	-H	255.0665,183.1029,117.0350,68.9984	20.54	C,L,P,R,S,X

74	Glaurin	$C_{16}H_{32}O_4$	0.98	288.23034	287.22306	-H	288.2263,287.2231,141.1286	10.98	C,F,L,P,R,S,X
75	Byakangelicol	$C_{17}H_{16}O_6$	1.74	316.09524	315.08796	-H	231.0287,214.0629,201.0561,95.0143	2.67	F,L,P,S
76#	Paeoniflorigenone	$C_{17}H_{18}O_6$	0.83	318.1106	319.11788	+H	319.1366,151.0756,105.0336	14.69	C,L,P,R,S,X
77#	Paeonilactone C	$C_{17}H_{18}O_6$	0.47	318.11049	319.11776	+H	105.0335,77.0386,54.3831	7.44	C,F,L,P,R,S,X
78#	4-hydroxypaeoniflorigeno	$C_{17}H_{18}O_7$	1.29	334.10568	335.11297	+H	335.1671,303.1417,121.0285	13.56	C,F,R
79	Mycophenolic acid	$C_{17}H_{20}O_6$	1.13	320.12635	319.11907	-H	319.1190,275.1297,191.0352	1.49	C,R
80	Matricin	$C_{17}H_{22}O_5$	1.64	306.14723	305.13995	-H	305.0309,245.9855,219.4317,125.0248	21.14	C,L,P,R,S,X
81	Loganin	$C_{17}H_{26}O_{10}$	1.11	390.15303	389.14575	-H	343.1400,227.8675,109.0660	1.88	C,L,R,S
82	Embelin	$C_{17}H_{26}O_4$	1.78	294.18363	293.17636	-H	141.1295,139.0409	21.58	C,L,P,R,S,X
83	Monomyristin	$C_{17}H_{34}O_4$	1.28	302.2461	301.23882	-H	181.0883,179.0723,147.0457,73.8200,58.9876	9.28	C,F,L,P,R,S,X
84	Octinoxate	$C_{18}H_{26}O_3$	2.42	290.1889	289.18162	-H	289.0721,159.0455,133.0306,131.9848,129.9725	20.56	L,P,S
85	Di-n-Amyl phthalate	$C_{18}H_{26}O_4$	1.91	306.18369	305.17641	-H	305.1765,287.1662,135.1819,125.0975	21	C,L,P,R,S,X
86	Gamma-Linolenic acid	$C_{18}H_{30}O_2$	1.41	278.22497	277.21753	-H	277.9914,276.9886,164.7702	21.92	F,L,P,S
87	4-Dodecylbenzenesulfonic acid	$C_{18}H_{30}O_3S$	1.28	326.19198	325.18471	-H	325.1849,197.0271,184.0175,183.0124,170.0047	24.68	C,F,L,P,R,S,X
88	2,3-dinor Prostaglandin E1	$C_{18}H_{30}O_5$	2	326.20998	325.20271	-H	307.1921,209.1186,325.2033	16.79	L
89	Oleic acid	$C_{18}H_{34}O_2$	1.11	282.25619	281.24892	-H	282.9539,264.9448,263.9545	22.37	C,S,X
90	Octadecanedioic acid	$C_{18}H_{34}O_4$	1.63	314.24622	313.23895	-H	57.6250,56.5988	21.19	X
91	Sphingosine	$C_{18}H_{37}NO_2$	0.79	299.28267	300.28994	+H	300.2899,283.2627,282.2780,95.0857	23.24	C,L,R,S,X
92	Phytosphingosine	$C_{18}H_{39}NO_3$	2.55	317.2938	318.30108	+H	318.3012,300.290,160.2490	19.23	C,F,L,P,R,S,X
93	Aloesin	$C_{19}H_{22}O_9$	1.59	394.12701	393.11973	-H	393.0461,273.8029,203.0340,171,0194,161.0453	10.58	L,P,S
94	Sucrose 6-benzoate	$C_{19}H_{26}O_{12}$	1.07	446.1429	445.13552	-H	184.9547,127.0405	10.1	C,R
95	1'-O-galloylsucrose	$C_{19}H_{26}O_{15}$	1.54	494.12793	495.13519	+H	330.9870,227.7980,133.9352,123.8773	4.41	F,L
96	Methyl octadecadienoate	$C_{19}H_{34}O_2$	0.24	294.25595	295.26323	+H	295.2630,111.1167,69.0698	21.25	P,R,S,X
97	Methyl linoleate	$C_{19}H_{34}O_2$	0.24	294.25595	295.26323	+H	95.0852,83.0852,81.0695,69.0698,55.0541	22.15	C,L,P,R,S,X
98#	Juglalin	$C_{20}H_{18}O_{10}$	1.74	418.09072	417.08341	-H	417.0833,284.0330,255.0304	14.64	C,L,P,R,S
99	Maltitol	$C_{12}H_{24}O_{11}$	-0.23	344.13178	343.12449	-H	343.1147,179.0559,101.0245,89.0244,71.0139	1.18	F,L,P,S
100	Lauric acid	$C_{12}H_{24}O_2$	0.64	200.17776	199.17048	-H	200.0412,199.8514,73.8640	22.14	P,S
101#	Quercitrin	$C_{21}H_{20}O_{11}$	2.52	448.10169	447.09441	-H	448.0787,447.0840,300.4267,269.0459	11.38	C,R,S,X
102	Trifolin	$C_{21}H_{20}O_{11}$	1.37	448.10169	447.0939	-H	447.0936,284.0327,255.0300	10.10	F,L,P,S
103	Luteolin-7-glucoside	$C_{21}H_{20}O_{11}$	-0.13	448.10169	449.10778	+H	329.0658,287.0550,153.0183,85.0283	7.08	F,L,P,S
104	Isoquercetin	$C_{21}H_{20}O_{12}$	0.79	464.09584	463.08856	-H	463.0875,301.0707,271.0611,151.0400,100.9333	11.76	F
105	Myricitrin	$C_{21}H_{20}O_{12}$	0.78	464.09584	463.08859	-H	301.0350,300.0279,271.0251	9.36	C,F,L,P,R,S
106	Hyperin	$C_{21}H_{20}O_{12}$	0.75	464.09584	465.10309	+H	465.2316,304.0534,303.0501	9.4	C,F,L,P,S
107#	Prunin	$C_{21}H_{22}O_{10}$	2.19	434.12225	435.12948	+H	434.1170,433.1162	18.91	C,R,X
108	Astilbin	$C_{21}H_{22}O_{11}$	2.42	450.1173	449.11002	-H	449.1093,297.0614,151.0403	11.43	L,P,S
109#	Eriodictyol-7-O-glucoside	$C_{21}H_{22}O_{11}$	0.88	450.11661	449.10929	-H	449.1102,287.0566,151.0038,107.0142	13.09	F,R

110	Plantagoside	$C_{21}H_{22}O_{12}$	2.06	466.11209	465.10481	-H	465.1043,285.0408,125.0246	8.69	C,R,X
111	Phloridzin	$C_{21}H_{24}O_{10}$	2.7	436.13813	435.13063	-H	435.2193,273.0771,167.0351	10.9	C,F,R
112#	(+)-Catechin-5-O-glucoside	$C_{21}H_{24}O_{11}$	1.83	452.13269	451.1254	-H	451.1261,289.0722,271.0607,179.0353,177.0545	10.4	L,P,S
113	Atherosine	$C_{21}H_{32}O_{11}$	1.92	460.19535	505.19355	+HCOO	293.0883,165.0924,89.0245	6.9	C,R,X
114#	8-O-Isovaleryldes benzoylpaeoniflorin	$C_{21}H_{32}O_{11}$	-3.44	460.19535	461.20016	+H	461.2003,462.2042	16.05	F
115	Carboprost	$C_{21}H_{36}O_5$	1.84	368.25695	413.25517	+HCOO	413.1514,116.9289,100.9339	21.29	L
116	L-asparagine	$C_4H_8N_2O_3$	0.54	132.05356	133.06084	+H	116.0343,133.0609,87.0553,74.0237	2.53	L,P,S
117#	(-)-epicatechin-3-O-gallate	$C_{22}H_{18}O_{10}$	0.63	442.09028	443.09755	+H	153.0183,147.0443,139.0390,125.0237,123.0441	9.34	C,R,X
118#	Isorhamnetin-3giucoside	$C_{22}H_{22}O_{12}$	0.99	478.1116	477.10436	-H	315.0493,314.043,285.0406,271.0250,243.0301	10.1	C,F,L,P,R,S
119#	Pyridylpaeoniflorin	$C_{22}H_{27}NO_{11}$	0.92	481.15885	482.16614	+H	320.1134,179.0706,124.0394,85.0284	10.58	C,R,X
120#	Palbinone	$C_{22}H_{30}O_4$	1.24	358.21485	357.20758	-H	357.2075,358.2108,301.2181,243.1757	18.22	C,R,S,X
121	Ascorbyl palmitate	$C_{22}H_{38}O_7$	0.84	414.2621	413.25482	-H	327.1829,87.0091,71.0140,59.0139,56.3758	21.13	S
122#	Paeonin B	$C_{23}H_{28}O_{11}$	0.79	480.16354	481.17082	+H	197.0811,155.0335,151.0755,133.0648	7.43	C,F,L,P,R,S,X
123#	Mudanpioside D	$C_{24}H_{30}O_{12}$	2.33	510.17491	509.16764	-H	479.7846,165.0559	9.46	C,F,L,P,R,S,X
124#	Mudanpioside E	$C_{24}H_{30}O_{13}$	1	526.16917	525.16189	-H	363.1099,345.0980,327.0921,167.0351,165.0557	7.98	C,F,L,P,R,X
125#	Kaempferol-3,7-di-o-glucoside	$C_{27}H_{30}O_{16}$	2.08	610.15465	609.14738	-H	447.0935,285.0408,283.0252,255.0303	7.05	F,L,P,S
126#	Quercetin 3-O-robinobioside	$C_{27}H_{30}O_{16}$	1.44	610.15426	611.16154	+H	304.0543,303.0504	7.05	F,L,P,S
127#	Quercetin 3,7-diglcoside	$C_{27}H_{30}O_{17}$	2.04	626.14958	625.14231	-H	505.1002,463.0878,301.0356	6.34	F,L,P,S
128	Turkesterone	$C_{27}H_{44}O_8$	0.72	496.30397	497.31125	+H	197.0810,133.0650,121.0287	11.29	C,F,R
129#	Isorhamnetin 3,7-O-diglucoside	$C_{28}H_{32}O_{17}$	0.71	640.1644	639.15713	-H	179.0562,161.0458,101.0245	15.71	F,L,P,S
130#	6-O-β-D-Glucopyranosylalbiflorin	$C_{29}H_{38}O_{16}$	1.37	642.21686	641.20939	-H	641.2070,611.2024,489.1623,323.0991,121.0297	12.87	F
131	Dodecanedioic acid	$C_{12}H_{22}O_4$	0.98	230.15203	229.14476	-H	229.1446,211.1341,167.1441	10.06	P,S
132#	Isomaltopaeoniflorin	$C_{29}H_{38}O_{16}$	1.02	642.21664	643.22392	+H	197.0810,151.0749,133.0647,105.0335	7.34	L,P,S
133	Procyanidin B1	$C_{30}H_{26}O_{12}$	-0.11	578.14236	579.14964	+H	407.0848,289.0331,245.0445,161.0598	5.35	C,R,X
134#	Paeonin A	$C_{30}H_{32}O_{12}$	0.07	584.18942	585.19672	+H	249.0756,197.0805,105.0334	14.05	C,F,L,P,R,S,X
135#	Mudanpioside H	$C_{30}H_{32}O_{14}$	1.68	616.18024	615.17296	-H	615.1673,493.1355,169.0147	20.95	C,F,L,P,R,S,X
136#	6'-O-galloylalbiflorin	$C_{30}H_{32}O_{15}$	1.5	632.17507	633.18134	+H	315.0718,153.0184,105.0336	9.36	C,F,L,R,X
137#	Galloyloxypaeoniflorin	$C_{30}H_{32}O_{16}$	0.92	648.16963	649.17694	+H	136.6436,124.7324	18	C,F,R
138#	Oleanolic aldehyde	$C_{30}H_{48}O_2$	3.52	440.36698	441.37426	+H	151.1326,135.1172,133.1018,121.1013	21.94	C,P,R,S,X
139#	Ursolic acid	$C_{30}H_{48}O_3$	1.72	456.36113	455.35383	-H	456.3572,455.3536,453.1761	23.87	C,P,R,S
140#	Mudanpioside B	$C_{31}H_{34}O_{14}$	1.78	630.19598	629.1887	-H	456.3572,455.3536	22.39	C,F,L,P,R,S
141	Tellimagrandin I	$C_{34}H_{26}O_{22}$	0.21	786.09174	785.0848	-H	535.1622,431.1356,165.0557,151.0761	7.02	C,R
142#	1,2,3,6-Tetra-O-galloyl-β-D-glucose	$C_{34}H_{28}O_{22}$	1.31	788.10825	787.10097	-H	449.0738,169.0144,153.0198,123.0089,101.0247	8.81	C,F,L,P,R,S,X
143#	Procyanidin B2-3''-O-gallate	$C_{37}H_{30}O_{16}$	1.58	730.15454	731.16182	+H	275.0570,271.0609,153.0185,139.0393,123.0448	9.49	C,R,X
144#	3’,6’-Di-O-galloylpaeoniflorin	$C_{37}H_{36}O_{19}$	1.76	784.18646	785.19376	+H	305.0294,153.0185,105.0336	22.05	C,F,R,X
145	Maleic acid	C_4H4O_4	0.71	116.01104	115.00376	-H	115.0039,72.0176,71.0139	0.69	C,F,L,P,R,S,X

146	Fumaric acid	C_4H4O_4	1.07	116.01108	115.00381	-H	115.0410,114.9332,97.9314,71.0139,45.2400	1.16	C,F,L,P,R,S,X
147	2-Oxobutyric acid	$C_4H_6O_3$	0.39	102.03173	101.02445	-H	101.9335,99.9259,57.0346,55.0190	0.86	C,L,P,R,S,X
148	D-(+)-Malic acid	$C_4H_6O_5$	0.67	134.02161	133.01433	-H	133.0143,115.0038,89.0245,72.9932,71.0139	0.86	C,L,P,R,S,X
149	L-(-)-Malic acid	$C_4H_6O_5$	0.64	134.02161	133.01433	-H	133.0143,115.0038,89.0244,72.9932,71.0139	1.16	F,L,P,R,S
150	Casuarinin	$C_41H_{28}O_{26}$	-0.14	936.08674	937.5868	+H	375.1984,155.0705,137.0600,125.0603	24.04	C,P,S
151	2-Furoic acid	C_5H4O_3	0.69	112.01612	111.00884	-H	111.0089,110.9865,83.0140,67.0189	0.84	C,F,R,S,X
152	Trans-Glutaconic acid	$C_5H_6O_4$	-0.52	130.02654	131.03385	+H	113.0345,95.0126,85.0283,71.0320,57.0331	0.78	C,R,S,X
153	L-Proline	$C_5H_9NO_2$	-0.65	115.06325	116.07053	+H	116.0705,70.0650,41.5960	0.8	C,F,L,P,R,S,X
154	Adipic acid	$C_6H_{10}O_4$	1.32	146.0581	145.05082	-H	145.0507,127.0402,101.0609,83.0504,81.0346	0.77	C,S,X
155	D-Saccharic acid	$C_6H_{10}O_8$	0.1	210.03759	209.03031	-H	209.0303,191.0198,85.0295,59.0139,57.0346	0.83	C,F,L,P,R,S,X
156	α-hydroxyisocaproic acid	$C_6H_{12}O_3$	1.52	132.07885	133.08612	+H	69.0339,57.9428,43.0179,41.0389	16.79	C,F,R,X
157	Catechol	$C_6H_6O_2$	0.78	110.03687	109.02959	-H	109.0296,108.0219,91.9317,81.0345	1.19	F,P,S
158	Pyrogallol	$C_6H_6O_3$	0.99	126.03182	125.02454	-H	125.0245,97.0295,81.0347,79.0191,69.0346	12.57	C,F,L,R,X
159	Trans-Aconitic acid	$C_6H_6O_6$	0.26	174.01648	173.00921	-H	173.0092,129.0193,111.0450,85.0294	2.6	F,L,P,S
160	Cis-Aconitic acid	$C_6H_6O_6$	0.32	174.01649	173.00922	-H	172.0987,129.0194,111.0089,85.0296	0.71	C,R,S,X
161	Isocitric acid	$C_6H_8O_7$	0.15	192.02703	191.01976	-H	191.0561,173.0459,128.0440	1.2	C,F,L,P,R,S,X
162	Citric acid	$C_6H_8O_7$	0.45	192.02709	191.01981	-H	173.0090,130.9987,129.0194,111.0088,87.0088	2.25	C,F,L,P,R,S,X
163	L-Ascorbic acid 2-sulfate	$C_6H_8O_9S$	-2.07	255.98837	254.9811	-H	138.1932,80.1195,71.4448,59.2311	16.82	F
164	D-(-)-Quinic acid	$C_7H_{12}O_6$	-0.22	192.06335	191.05608	-H	191.0561,93.0347,85.0295	4.58	F,L,P,S
165	Benzaldehyde	C_7H_6O	0.03	106.04187	107.04914	+H	107.0491,106.0734,79.0542	8.26	C,F,R,X
166	4-Hydroxybenzaldehyde	$C_7H_6O_2$	1.58	122.03697	121.0297	-H	121.0297,93.0353,92.0269	10.16	C,F,L,P,R,S,X
167#	Salicylic acid	$C_7H_6O_3$	1.02	138.03183	137.02456	-H	137.0244,93.0347	5.33	C,F,L,P,R,S
168	3-Hydroxybenzoic acid	$C_7H_6O_3$	1.85	138.03195	137.02467	-H	137.0246,93.0347,44.6557	10.58	C,L,P,R,S,X
169	4-Methylphenol	C_7H_8O	1.28	108.05765	107.05038	-H	94.0412,66.0465,65.0384	2.5	L
170	P-Xylene	C_8H_{10}	-0.24	106.07822	107.0855	+H	107.0855,105.0700,91.0543,77.0388	7.95	C,P,R,S,X
171	Swainsonine	$C_8H_{15}NO_3$	-0.85	173.10505	174.1125	+H	156.1022,96.0802	0.84	C,R,S,X
172	5-methyl-2-heptanone	$C_8H_{16}O$	1.23	128.12027	127.0407	-H	127.0411,108.6596,83.9307,55.0193,41.9860	2.56	F,R
173	Phthalic anhydride	$C_8H_4O_3$	1.84	148.01632	149.02359	+H	149.0235,121.0285,93.0334,65.0386	8.16	C,L,R,X
174	Phthalic acid	$C_8H_6O_4$	1.32	166.02683	165.01955	-H	165.0559,121.0293,58.3710	24.69	P,R,S
175	Styrene	C_8H_8	1.1	104.06272	105.06997	+H	105.0701,87.0441,86.9529,59.0492	7.99	C,R,X
176	2-Methylbenzoic acid	$C_8H_8O_2$	1.65	136.05265	135.04538	-H	135.0454,134.8949,94.0381,93.0348	8.05	C,P,S
177	2-Anisic acid	$C_8H_8O_3$	1.14	152.04752	151.04024	-H	136.0531,107.0503,95.0503,59.0137,44.9982	2.55	L
178	3-Methoxysalicylic acid	$C_8H_8O_4$	0.94	168.04242	167.03514	-H	152.0118,123.0454,108.0218	8.48	F,S
179	4-Ethylphenol	$C_8H_{10}O$	0.14	122.07318	123.08046	+H	122.0967,108.0571,107.0484	7.99	L,R,X
180	Desaminotyrosine	$C_9H_{10}O_3$	1.82	166.0633	211.06148	+HCOO	149.0251,121.0300,73.7412,58.3780	11.73	R,S,X
181	Acetovanillone	$C_9H_{10}O_3$	0.06	166.063	167.07028	+H	167.0704,149.0598,121.0648	15.81	C,P,R,X

182	Dihydrocaffeic acid	$C_9H_{10}O_4$	0.77	182.05805	181.05077	-H	181.0725,121.0297,109.0154,59.0140	2.47	L
183	Syringic acid	$C_9H_{10}O_5$	0.63	198.05295	197.04567	-H	197.0457,182.0223,166.9988,123.0089	16.76	F,R,X
184#	Ethyl gallate	$C_9H_{10}O_5$	1.75	198.05317	199.06045	+H	169.2114,125.0238,124.1126	16.74	C,F,L,R,S
185	Uridine	$C_9H_{12}N_2O_6$	0.44	244.06964	243.06237	-H	243.0622,200.0565,153.0304,140.0354,110.0248	3.71	F,P,R,S,X
186	Pantothenic acid	$C_9H_{17}NO_5$	0.63	219.11081	218.10353	-H	218.1037,146.0824,111.9463,88.0405	1.92	C,F,R,S,X
187	Sistolynone	$C_9H_6O_2$	0.3	146.03682	147.0441	+H	147.0442,119.0492,91.0542	11.33	F,L,P,R,S
188	Trans-cinnamic acid	$C_9H_8O_2$	1.42	148.05264	147.04537	-H	147.0453,103.0555,102.9332,100.9336	14.12	L,P,S
189	3,4-dihydroxyphenylpyruvic acid	$C_9H_8O_5$	0.78	196.03733	195.03005	-H	177.0562,149.0606,123.0453,121.0296,109.0256	0.8	C,F,L,P,R,S,X
190	Tetralin	$C_{10}H_{12}$	-0.24	132.09387	133.10115	+H	117.1624,115.0546,104.0577	8	C,F,L,R,X
191#	(+)-paeonilactone B	$C_{10}H_{12}O_4$	1.15	196.07378	197.08106	+H	137.0599,123.080,107.0493	2.6	F,L,P,S
192#	Propyl gallate	$C_{10}H_{12}O_5$	1.06	212.0687	211.0614	-H	211.0614,167.0712,125.0248,124.0165	2.6	L
193#	Isopropylgallate	$C_{10}H_{12}O_5$	1.51	212.06879	211.06152	-H	167.0716,123.0617,81.0711	13.24	L
194	Butylbenzene	$C_{10}H_{14}$	1.68	134.10978	135.11705	+H	105.0700,92.0574,91.0543	17.79	C,F,L,P,R,S,X
195	Dyphylline	$C_{10}H_{14}N_4O_4$	-4.46	254.10037	253.0931	-H	255.0665,132.0583,123.0818	19.39	L
196	Myrtenal	$C_{10}H_{14}O$	0.32	150.10451	151.11177	+H	108.0883,107.0855,79.0542,41.0385	16.21	C,F,R,X
197	DL-carvone	$C_{10}H_{14}O$	0.32	150.10451	151.11177	+H	151.1118,123.0808,109.0645	12.68	C,F,R,X
198	Paeonisuffrone C	$C_{10}H_{14}O_2$	1.34	166.0996	167.10688	+H	167.0699,109.0648,106.9641	15.87	C,P,R,X
199	6-Amyl-2-pyrone	$C_{10}H_{14}O_2$	1.34	166.0996	167.10688	+H	167.1068,97.1011,93.0699,81.0699,67.0542	8.24	L,P,S
200	Mephenesin	$C_{10}H_{14}O_3$	0.76	182.09443	181.08715	-H	182.0906,133.0669,109.0661	1.2	C,F,P,S
201	Deoxypaeonisuffrone	$C_{10}H_{14}O_3$	-0.17	182.09426	183.10154	+H	183.1015,165.0911,109.0647,55.0179	3.16	C,L,P,R,X
202	(-)-Camphanic acid	$C_{10}H_{14}O_4$	1.02	198.08941	243.08763	+HCOO	179.0718,122.0375,85.0295,55.0346	1.45	C,R,X
203	Perillylalcohol	$C_{10}H_{16}O$	2.39	152.12048	135.11718	-OH	97.0653,81.0699,79.0544	21.93	C,L,P,R,S,X
204	D-camphor	$C_{10}H_{16}O$	2.39	152.12048	135.11718	-OH	107.085,105.0698,93.0699,91.0541,79.0543	23.55	C,L,P,R,S,X
205	Pinonic acid	$C_{10}H_{16}O_3$	1.07	184.11014	183.10287	-H	71.6030,68.8555,43.5819	5.42	C,F,L,P,R,S,X
206	Decarestrictine J	$C_{10}H_{16}O_4$	-0.34	200.10479	201.11207	+H	218.9840,190.9892,200,9736,84.9599	1.18	C,R,X
207	L-Glutathione (reduced)	$C_{10}H_{17}N_3O_6S$	0.08	307.08383	306.07656	-H	306.0767,272.0891,141.0670,128.0354	2.98	F
208	Sinapinic acid	$C_{11}H_{12}O_5$	0.87	224.06867	223.06139	-H	223.0616,208.0380,193.0143,179.0720,164.0479	0.81	C,F,R,S,X
209	3-Indoleacrylic acid	$C_{11}H_9NO_2$	1.43	187.0636	188.07086	+H	189.0747,188.0709,171.0638,170.0600	3.47	F,L,P,R,S,X
210	3-carboxy-4-methyl-5-propyl-2-furanpropionic acid	$C_{12}H_{16}O_5$	1.5	240.10013	239.09286	-H	221.0828,196.1057,195.1030,151.0765	5.51	L
211	Arbutin	$C_{12}H_{16}O_7$	1.78	272.09009	271.08281	-H	271.2283,145.0299,108.9931,93.0348,56.9440	14.46	C,F,L,P,R,S,X
212	3,8,9-trihydroxy-10-propyl-3,4,5,8,9,10-heXahydro-2H-oxecin-2-one	$C_{12}H_{20}O_5$	1.21	244.13137	243.12409	-H	243.1240,225.1134,207.1030	10.86	L,P,S
213	α,α-Trehalose	$C_{12}H_{22}O_{11}$	-0.09	342.11618	341.1088	-H	179.0563,161.0460,149.0457,143.0350,89.0345	0.77	C,F,L,P,R,S,X
214#	Peony ketone-1-O-β-D-glucoside	$C_{16}H_{24}O_9$	0.17	360.14209	359.13473	-H	359.1356,197.0814,179.0715	1.48	C,R,X

Note: *identified with a standard compound. #chemicals reported for the first time in *P. lactiflora* (45 in total). C denotes cortex in root, F flower, L leaf, P petiole, R root, S stem, X xylem in root.

Supplementary material 5 Identification of paeoniflorin based on automatic annotation by Compound Discoverer and manual annotation based on MS fragmentation

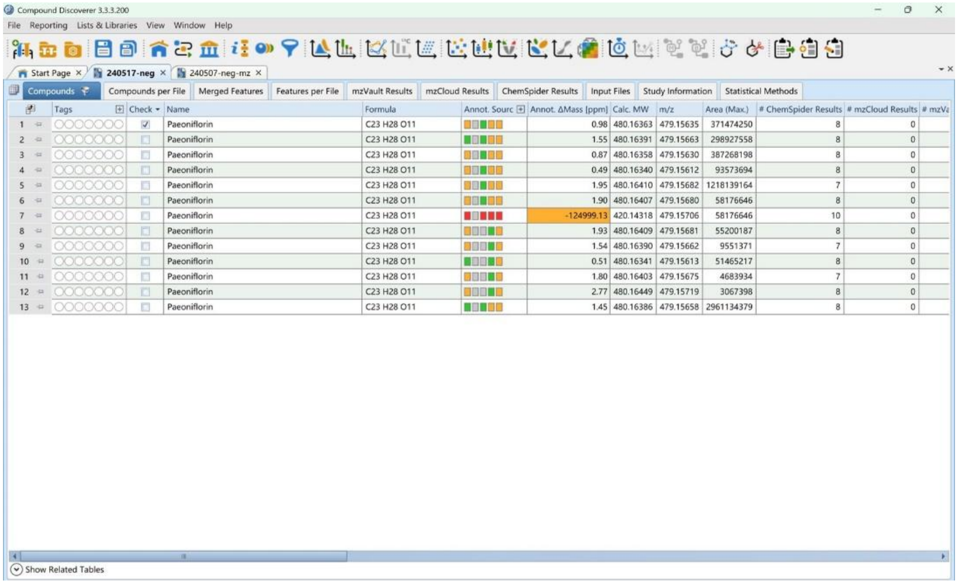

	Check	Name	Formula	Annot. ΔMass [ppm]	Calc. MW	m/z	Area (Max.)	# ChemSpider Results	# mzCloud Results
1	✓	Paeoniflorin	C23 H28 O11	0.98	480.16363	479.15635	371474250	8	0
2		Paeoniflorin	C23 H28 O11	1.55	480.16391	479.15663	298927558	8	0
3		Paeoniflorin	C23 H28 O11	0.87	480.16358	479.15630	387268198	8	0
4		Paeoniflorin	C23 H28 O11	0.49	480.16340	479.15612	93573694	8	0
5		Paeoniflorin	C23 H28 O11	1.95	480.16410	479.15682	1218139164	7	0
6		Paeoniflorin	C23 H28 O11	1.90	480.16407	479.15680	58176646	8	0
7		Paeoniflorin	C23 H28 O11	-124999.13	420.14318	479.15706	58176646	10	0
8		Paeoniflorin	C23 H28 O11	1.93	480.16409	479.15681	55200187	8	0
9		Paeoniflorin	C23 H28 O11	1.54	480.16390	479.15662	9551371	7	0
10		Paeoniflorin	C23 H28 O11	0.51	480.16341	479.15613	51465217	8	0
11		Paeoniflorin	C23 H28 O11	1.80	480.16403	479.15675	4683934	7	0
12		Paeoniflorin	C23 H28 O11	2.77	480.16449	479.15719	3067398	8	0
13		Paeoniflorin	C23 H28 O11	1.45	480.16386	479.15658	2961134379	8	0

Automatic annotation by Compound Discoverer

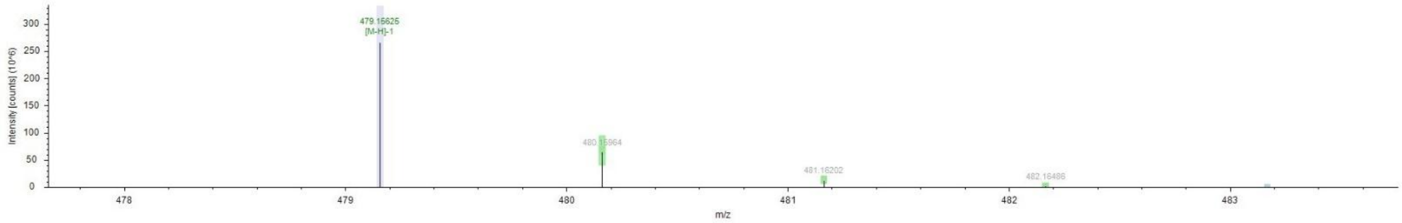

Parent Ion Information

Parent ion: m/z 479.1563 (negative ion mode, $[M-H]^-$), corresponding to a molecular weight of approximately 480.16 Da, which matches paeoniflorin ($C_{23}H_{28}O_{11}$, theoretical molecular weight 480.16 Da).

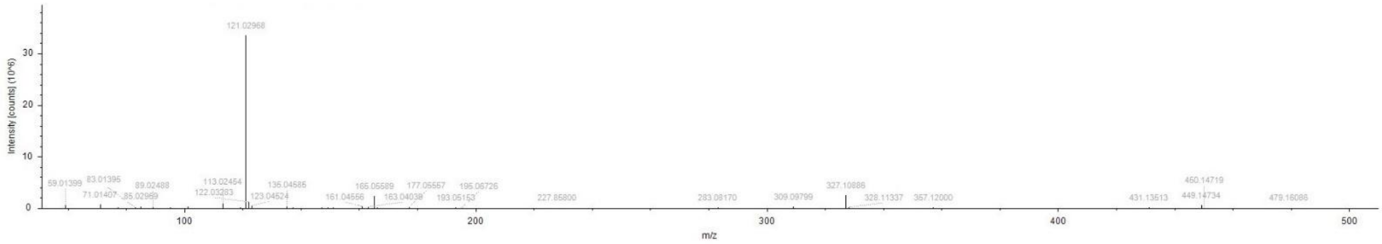

Characteristic Fragment Ions

Typical fragmentation pathway of paeoniflorin in negative ion mode and matching values:

m/z 449.14734: Likely attributed to the loss of CH_2O (sugar moiety cleavage), with an exact mass of 449.1447, corresponding to $C_{22}H_{25}O_{10}^-$.

m/z 327.10886: The actual fragment lost is $C_8H_8O_3^-$ (152.0473 Da), reflecting the synergistic cleavage of the benzoyl group and aglycone.

m/z 165.05589: The final fragment is $C_9H_9O_3^-$ (possibly a decarboxylation product), suggesting the stability of the monoterpene backbone.

m/z 121.02968 is a characteristic peak of paeoniflorin cleavage and should be the benzoic acid anion ($C_7H_5O_2^-$), reflecting the presence of the benzoyl group in the aglycone part, serving as a key basis for structural identification.
